# Enhancing cowpea wilt resistance: insights from gene coexpression network analysis with exogenous melatonin treatment

**DOI:** 10.1186/s12870-024-05289-w

**Published:** 2024-06-25

**Authors:** Yudi Gan, Zhiwei Tu, Youxin Yang, Liuyang Cheng, Nan Wang, Shuying Fan, Caijun Wu

**Affiliations:** College of Agronomy, Jiangxi Agricultural University, Nanchang, 330045 China

**Keywords:** Cowpea, Melatonin, *Fusarium oxysporum*, RNA-seq, WGCNA

## Abstract

**Background:**

Cowpea wilt is a harmful disease caused by *Fusarium oxysporum*, leading to substantial losses in cowpea production. Melatonin reportedly regulates plant immunity to pathogens; however the specific regulatory mechanism underlying the protective effect of melatonin pretreated of cowpea against *Fusarium oxysporum* remains known. Accordingly, the study sought to evaluate changes in the physiological and biochemical indices of cowpea following melatonin treated to facilitate *Fusarium oxysporum* resistance and elucidate the associated molecular mechanism using a weighted gene coexpression network.

**Results:**

Treatment with 100 µM melatonin was effective in increasing cowpea resistance to *Fusarium oxysporum*. Glutathione peroxidase (GSH-PX), catalase (CAT), and salicylic acid (SA) levels were significantly upregulated, and hydrogen peroxide (H_2_O_2_) levels were significantly downregulated in melatonin treated samples in roots. Weighted gene coexpression network analysis of melatonin- and *Fusarium oxysporum*-treated samples identified six expression modules comprising 2266 genes; the number of genes per module ranged from 9 to 895. In particular, 17 redox genes and 32 transcription factors within the blue module formed a complex interconnected expression network. KEGG analysis revealed that the associated pathways were enriched in secondary metabolism, peroxisomes, phenylalanine metabolism, flavonoids, and flavonol biosynthesis. More specifically, genes involved in lignin synthesis, catalase, superoxide dismutase, and peroxidase were upregulated. Additionally, exogenous melatonin induced activation of transcription factors, such as WRKY and MYB.

**Conclusions:**

The study elucidated changes in the expression of genes associated with the response of cowpea to *Fusarium oxysporum* under melatonin treated. Specifically, multiple defence mechanisms were initiated to improve cowpea resistance to *Fusarium oxysporum*.

**Supplementary Information:**

The online version contains supplementary material available at 10.1186/s12870-024-05289-w.

## Background

Cowpea (*Vigna unguiculata* L. Walp) is a significant legume vegetable with rich nutritional value, adaptability, and widespread cultivation [1, 2]. However, cowpea is also highly susceptible to epidemics caused by wilt [3]. *Fusarium oxysporum* is the main causal pathogen of cowpea wilt. This soil-borne pathogen can persist for extended periods due to its thick-walled spores. Classified as a hemibiotrophic pathogen, it can generate conidia and mycelia in response to root secretions from the host plant [4, 5]. Pathogens invade the roots of plants, colonize the vascular system, damage xylem vessels, disrupt the transportation of water and nutrients, and cause yellowing of leaves, ultimately resulting to withering or shedding of diseased leaves and the overall decline of the plant [6]. Due to the high rate of cowpea soil replanting, the prevalence of soil-borne diseases such as wilt disease has intensified. Eradication of cowpea wilt is a challenging task that requires careful management.

Melatonin is a hormone widely found in living organisms that has various regulatory functions in plant life, such as promoting root formation, fruit ripening, flowering, senescence, and alleviating biotic and abiotic stresses [7]. Hence, for plants to better adapt to stress and improve growth, researchers have extensively studied the application of melatonin under various stress scenarios. Melatonin attenuates the effects of abiotic stresses, such as cold stress [8], high temperature [9], drought [10], and salt stress [11]. It also increases resistance to cucumber downy mildew [12], cotton *Verticillium* wilt [13], tomato fruit grey mould [14], and potato *Phytophthora infestans* [15]. More specifically, melatonin enhances plant physiological functions, increases chlorophyII content, improves photosynthetic performance, and improves root capacity [16, 17]. Mechanistically, melatonin acts as a signalling molecule that mitigates the negative effects of various stresses by regulating the synthesis of phytohormones and physiological and biochemical substances, expression of resistance genes, and activation of plant defence systems [18].

Plants have evolved two innate immune systems to cope with pathogens. Plants can recognize pathogens through cell surface-localised pattern recognition receptors, which then activate of pathogen associated molecular patterns (PAMP)-triggered immunity, and initiate the effector-triggered immunity (ETI) by intracellular nucleotide-bound leucine-rich receptors (NLRs) [19, 20]. Reactive oxygen species (ROS) accumulation occurs monophasically and transiently during PAMP-triggered immunity responses and biphasically and continuously during ETI responses [21]. Meanwhile, increased ROS levels affect the function of peroxisome functions—multifunctional organelles that detoxify hydrogen peroxide (H_2_O_2_) through antioxidant enzymes such as glutathione peroxidase (GSH-PX), catalase (CAT), peroxidase (POD), and ascorbate peroxidase (APX) [22, 23]. Moreover, ROS are interconnected downstream and upstream with salicylic acid signalling [24]. Under stress conditions, salicylic acid participates in the antioxidant response via ascorbate glutathione [25]. Plants have also evolved biosynthetic mechanisms for secondary metabolites, such as phenylpropanes, terpenoids, and alkaloids, in response to biotic stress [26]. Transcription factors (TFs) families, such as WRKY and NAC, also contribute to immune responses [27]. For instance, CmWRKY6-1 and CmWRKY8-1 could enhance resistance to *Fusarium oxysporum* in chrysanthemum [28, 29], and NACL-D1 improves the resistance to Fusarium wilt in wheat [30].

Although the numerous roles played by melatonin in plant immunity have been established, the mechanism by which it induces resistance to Fusarium wilt in cowpea remains unclear. In this study, the physiological responses of cowpea treated with melatonin in response to *Fusarium oxysporum* were investigated, resulting in a significant alleviation of wilt symptoms. Transcriptional analyses were conducted to identify metabolic pathways associated with disease resistance in cowpea under various melatonin treatments following inoculation with *Fusarium oxysporum*. Subsequently, Weighted Gene Co-expression Network Analysis (WGCNA) was utilized to identify gene modules and hub genes linked to induction of resistance in melatonin-treated cowpea against *Fusarium oxysporum*. Elucidating these mechanisms will offer valuable insights into molecular basis of melatonin-induced resistance in cowpea against wilt, which can guide breeding efforts for disease resistance.

## Results

### Morphological and molecular identification of *Fusarium oxysporum*

**Fig. 1:**
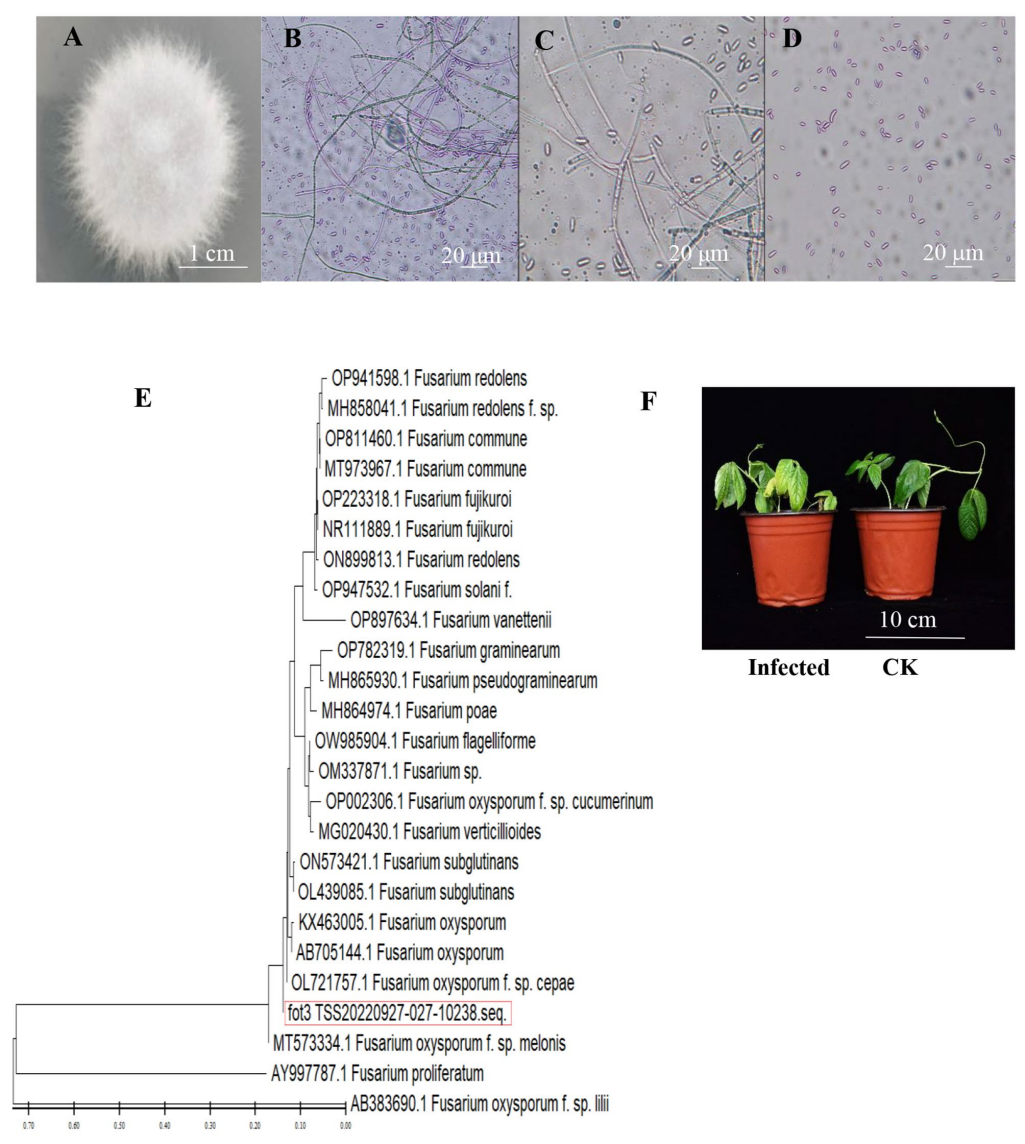
Identification of pathogenic bacteria (**A**) *Fusarium oxysporum* colony colony morphology. (**B**) Hyphas. (**C**) Conidiophores. (**D**) Micoconidia. (**E**) Phylogenetic tree of *Fusarium oxysporum* ITS gene sequence construction. (**F**) Cowpea infested with *Fusarium oxysporum* for 20 days

The colonies of the isolated *Fusarium oxysporum* were white flocculent mycelia and slightly pinkish-purple in the centre (Fig. 1A). The morphological characteristics of *Fusarium oxysporum* were consistent with previous findings [5]. *Cladosporium spp* exhibited septate and branched mycelium with a flask-shaped conidial peduncle. Small reniform or ovate conidia were observed, measuring about 5.01–11.52 μm × 1.41–2.21 μm, with no presence of large conidia (Fig. 1B-D). Molecular identification of *Fusarium oxysporum* was performed by PCR amplification, and the target fragments were obtained with the NCBI website BLAST tool. Phylogenetic tree construction showed that the sequences of the target fragments shared the highest similarity with OL721757.1, AB705144.1, and KX463005.1 (Fig. 1E). The OL721757 sequence was isolated from *Fusarium oxysporum* of onion tissues, and the AB7055144.1 sequence was isolated from *Fusarium oxysporum* of oilseed rape. Cowpea plants were assessed for pathogenicity by spore irrigation, revealing that the leaves lost water, wilted, yellowed, and withered 20 days post-inoculation (dpi) (Fig. 1F).

### Exogenous melatonin improves cowpea resistance to *Fusarium oxysporum*

**Fig. 2:**
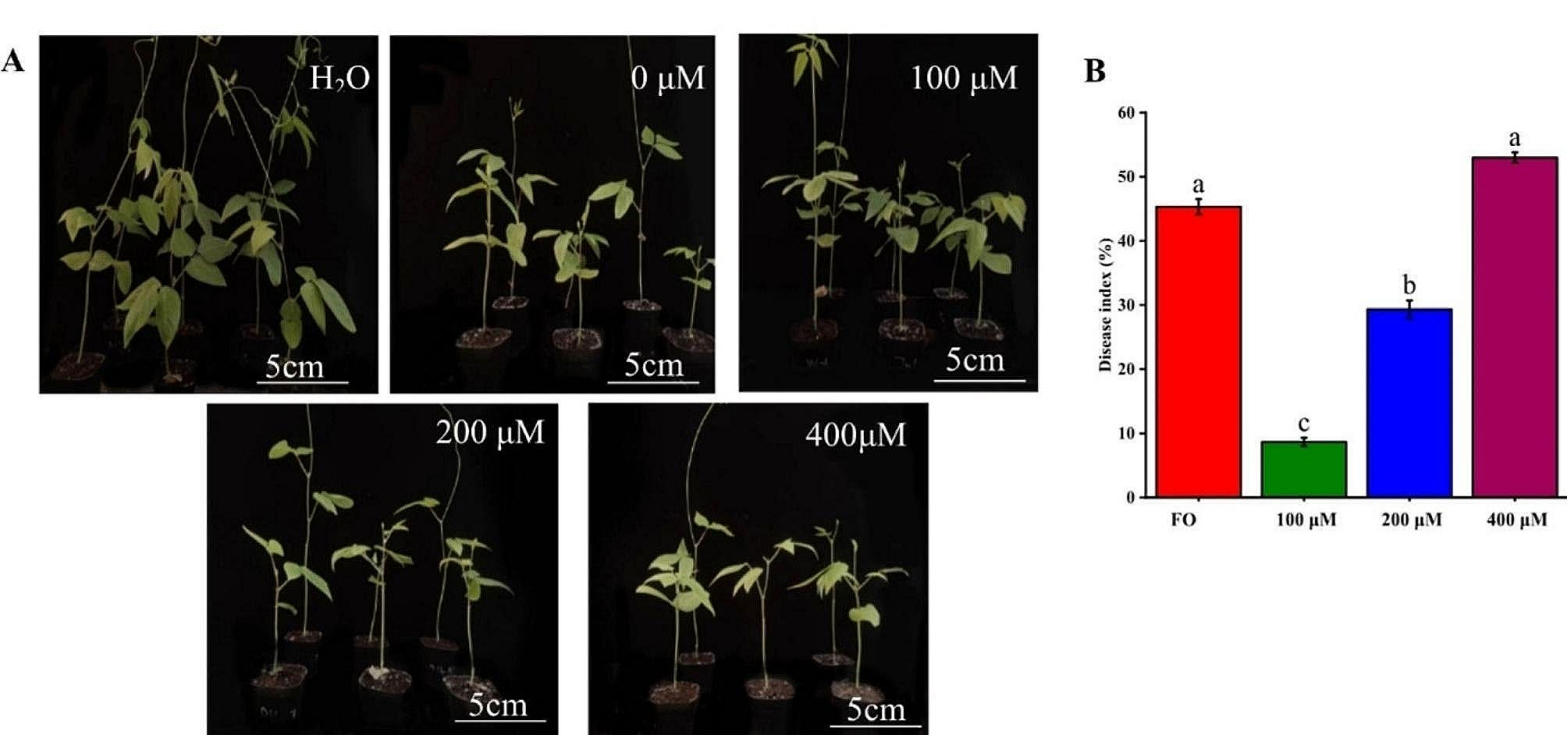
Effects of different exogenous melatonin concentrations on cowpea wilt disease. (**A**) Phenotypes of plants pretreated with different concentrations of exogenous melatonin at 20 days post-inoculation (dpi). Bar = 5 cm. (**B**) Disease index in each treatment group at 20 dpi

Cowpea seedlings underwent with 0, 100, 200, and 400 µM of melatonin treated (MT) (Fig. 2A). The control group (CK) grew normally and initiated vine development, whereas plants in the MT group demonstrated marked growth inhibition, characterised by dwarfism, yellowing, and reduced leaves. However, the suppression was relieved at 100 µM MT, showing the greatest decrease in disease index, approximately 37% (Fig. 2B). The disease index of 400 µM MT did not differ from that of *Fusarium oxysporum* treated (FO).

### Transcriptome analysis

**Fig. 3:**
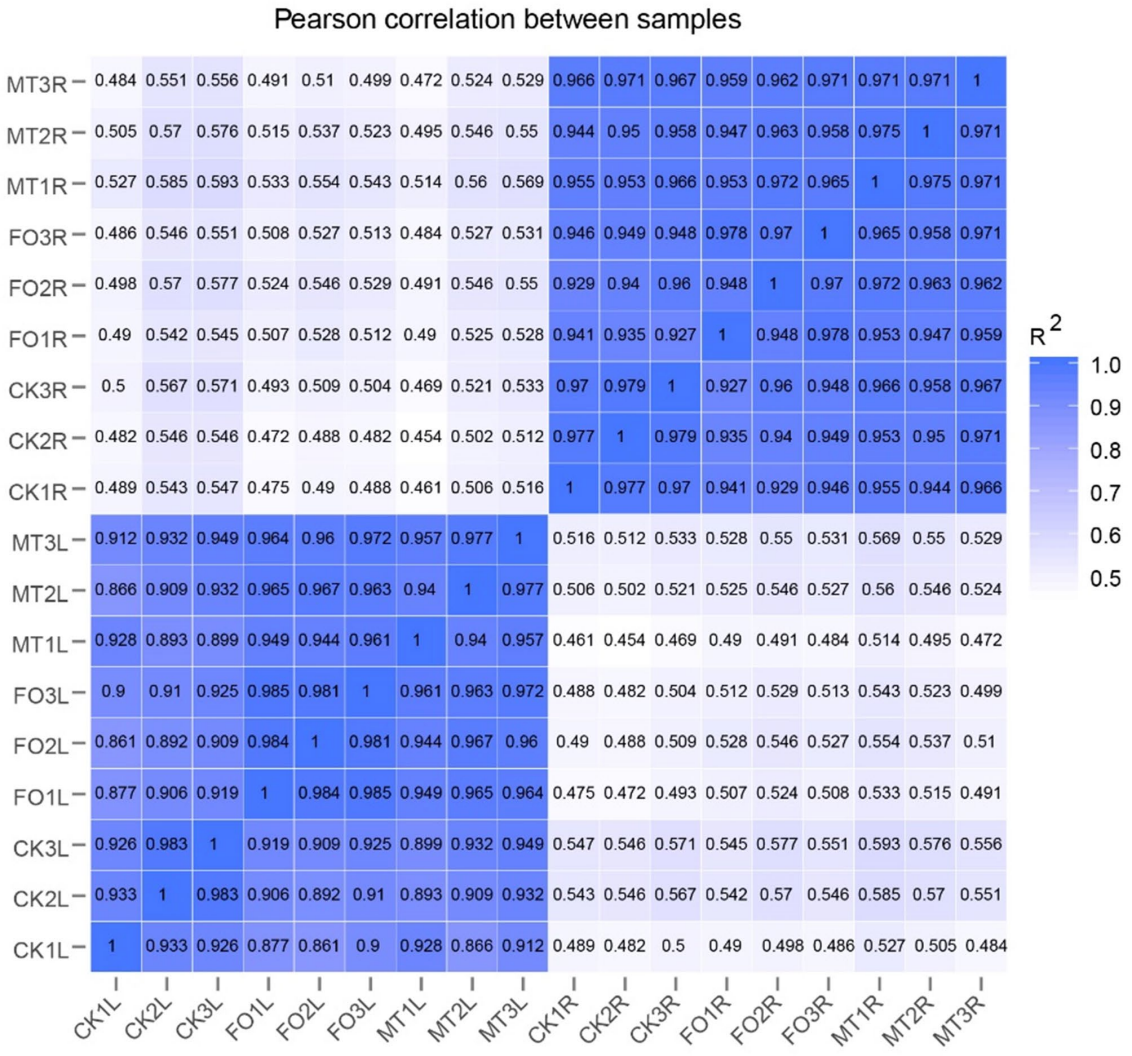
Pearson correlation coefficient in the sample

To investigate the regulatory mechanisms of MT on cowpea resistance to *Fusarium oxysporum*, differentially treated samples were analysed by RNA-seq. The root and leaf samples were categorised: control samples in leaves (CKL), control samples in roots (CKR), FO samples in leaves (FOL), FO samples in roots (FOR), melatonin treated samples in leaves (MTL), and melatonin treated samples in roots (MTR). A total of 85.5 G of clean reads were generated for all samples (Q30 ≥ 93.17%) (Additional file 1: Table S1). Pearson correlation coefficients (*r*^*2*^) ranged from 0.926 to 0.985, indicating high similarity of expression patterns between samples (Fig. 3).

### Differentially expressed genes in MT cowpeas under *Fusarium oxysporum* infestation

**Fig. 4:**
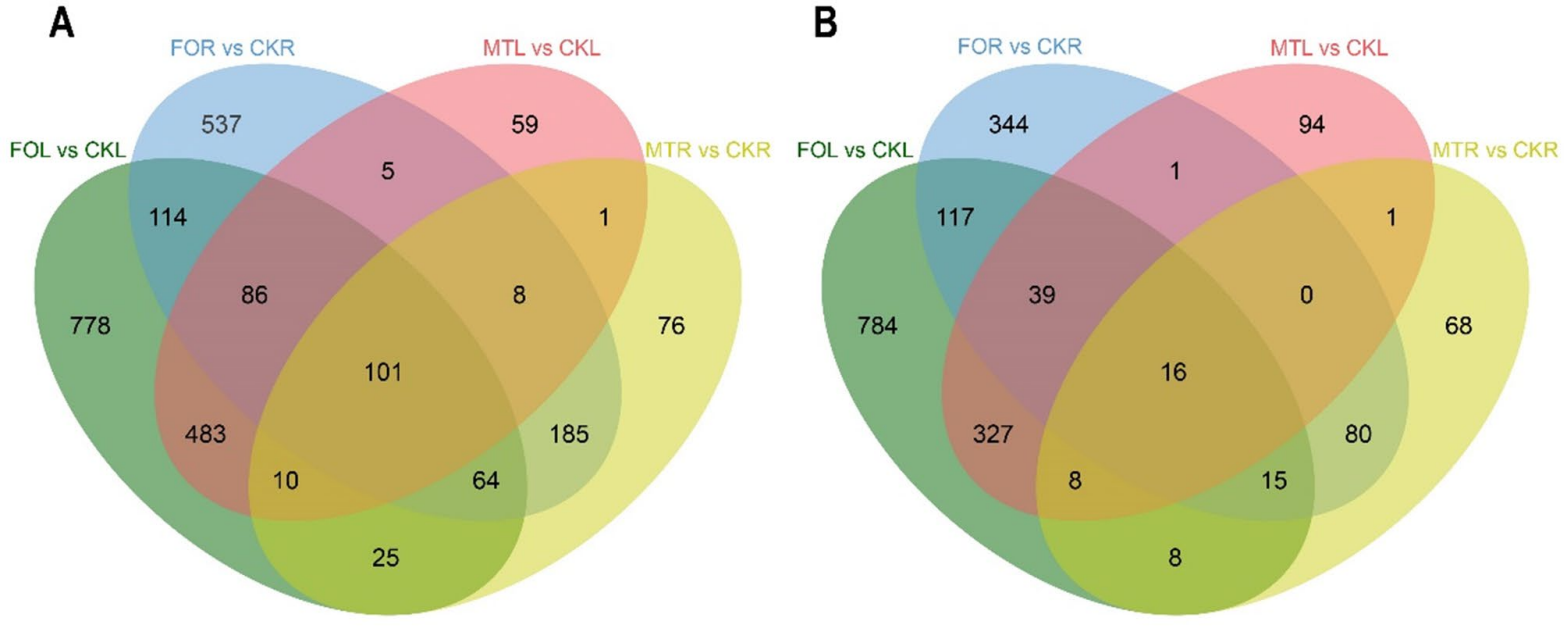
Differential genes expression analysis. (**A**) Upregulation genes in roots and leaves under FO and MT. (**B**) Downregulated genes in roots and leaves under FO and MT

In response to *Fusarium oxysporum*, melatonin treatment led to differentially expressed genes (DEGs) in cowpea. We compared the DEGs between treatments in both FOR and FOL. In FOL, a total of 2975 DEGs were identified, of which 1661 genes were upregulated in response to *Fusarium oxysporum* and 1314 genes were downregulated. In FOR, 1712 DEGs were identified, of which 1100 genes were upregulated. In the comparison between FOL and FOR, 552 DEGs were shared between the groups, 365 genes were positively expressed, and 187 genes were negatively expressed. In the comparison between MTL and MTR, 753 and 470 genes were positively regulated, while 486 and 196 genes were negatively regulated, respectively. MT induced the upregulation of 120 genes and the downregulation of 25 genes. Comparing the two treatments and the two tissues, most genes (101) were upregulated, while only 16 genes were downregulated (Fig. 4). Hence, the number of DEGs decreased significantly with MT.

Notably, we identified genes associated with plant defence among the DEGs. In particular, genes that were upregulated in the MTL included those associated with CYP73A100 (gene-LOC114189889) involved in the synthesis of *p*-coumaric acid, flavonol 3-O-methyltransferase (gene-LOC114192266, gene-LOC114182114), and 4-coumarate-CoA ligase (gene-LOC114185049). We also identified upregulated genes involved in the synthesis of mannitol dehydrogenase (gene-LOC114177535) and hydroxycinnamoyl transferase synthesis (gene-LOC114178886) in the MTR. Meanwhile, genes related to β-glucosidase (BGLU) synthesis (gene-LOC114186321, gene-LOC114187951, gene-LOC114169017) and lignin synthesis-associated POD (gene-LOC114188922, gene-LOC114171444) were upregulated in MTL and MTR.

### Gene expression pattern analysis

**Fig. 5:**
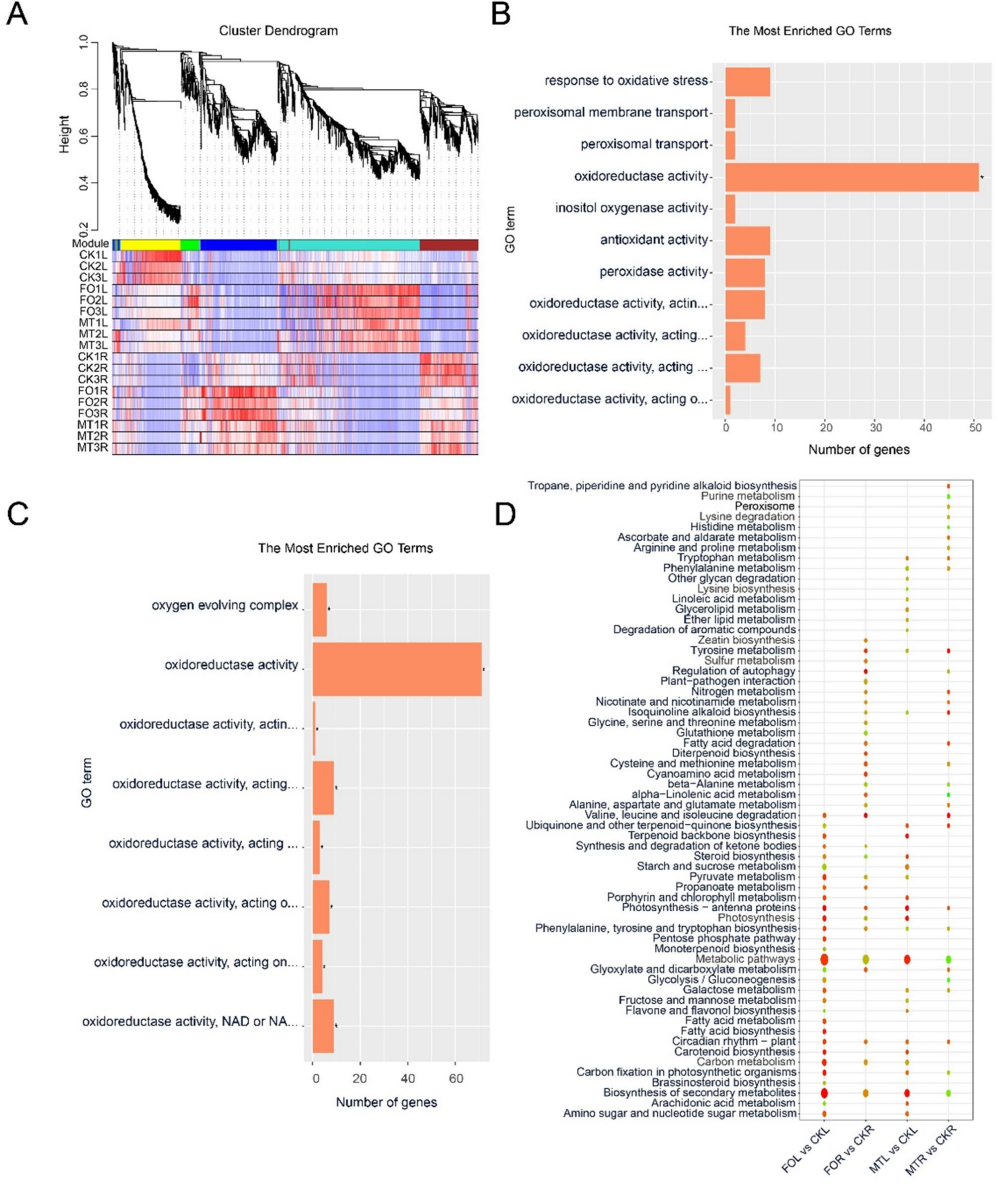
Gene network coexpression analysis (**A**) Gene expression module and expression level analysis. (**B**) GO enrichment of blue module genes. (**C**) GO enrichment of turquoise module genes. (**D**) Metabolic pathways enriched in roots and leaves under FO and MT treatments

To further characterise the effect of melatonin treatment on *Fusarium oxysporum* resistance, we performed WGCNA analysis. Six coexpressed gene modules were identified (Fig. 5A). A total of 2,266 genes were used for WGCNA, ranging from 9 to 895 DEGs in each module. In particular, most genes from the blue module (493 genes) were positively expressed in both the FOR and MTR groups, with 334 (67.75%) and 168 (34.08%) genes, respectively. Meanwhile, the turquoise module (895 genes) was significantly upregulated in FOL and MTL with 612 (68.38%) and 371 (41.45%) genes, respectively.

Many redox-related GO terms were identified in the blue and turquoise module genes (Fig. 5B, C). The blue module was enriched primarily in terms involved in redox reactions, including oxidoreductase activity, inositol oxygenase activity, oxidative stress, antioxidant activity, peroxisomal membrane transport, peroxidase activity, oxidoreductase activity (acting on peroxide), and peroxisomal transport. The turquoise module was enriched predominantly in oxidoreductase activity with similar terms.

The main metabolic pathways enriched by KEGG analysis were phenylalanine metabolism, photosynthesis, gluconeogenesis, secondary metabolism, peroxisomes, phenylalanine, tyrosine, and tryptophan biosynthesis pathways, flavone and flavonol biosynthesis, and tryptophan metabolism (Fig. 5D). In particular, peroxisomes were enriched only in the MTR. Isoquinoline alkaloid biosynthesis was enriched in the MTR. Phenylalanine and tryptophan metabolism were enriched only in MTR and MTL. Flavone and flavonol biosynthesis were enriched in the MTL. Secondary metabolite biosynthesis was enriched in the MTL and FOL groups (Additional File 2: Fig. S1). Plant-pathogen interactions were enriched in MTR and FOR (Additional file 2: Fig. S2).

### Expression module correlation analysis with indices

**Fig. 6:**
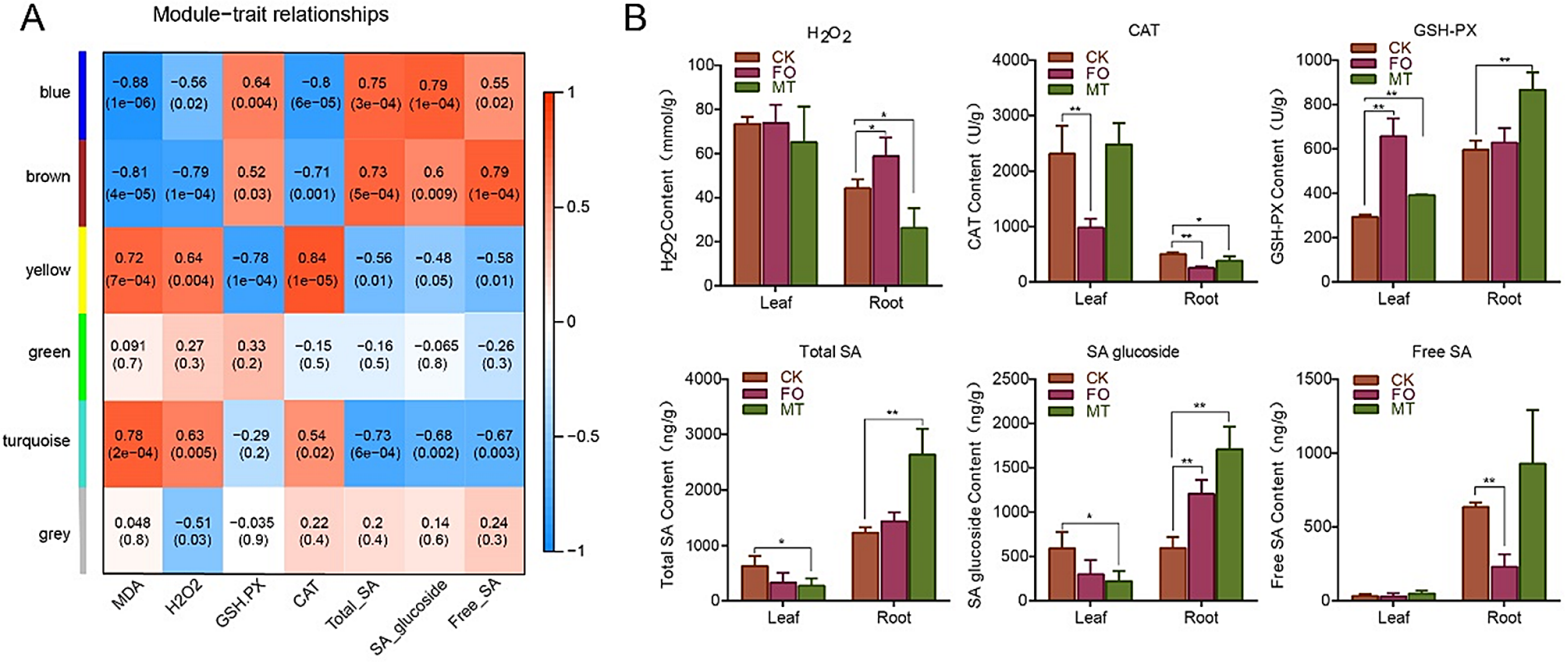
Expression module correlation analysis with indices (**A**) Gene expression module and indices association analysis. (**B**) Six biochemical phenotypes under two treatments. **P < 0.01; *P < 0.05

The levels of GSH-PX, total SA, and SA glucoside were significantly and positively correlated with the blue module (*r* = 0.64, *p*-value = 0.004; *r* = 0.75, *p*-value = 3e−04; *r* = 0.79, *p*-value = 1e−04) and inversely correlated with the turquoise expression module (*r* = −0.29, *p*-value = 0.2; *r* = −0.73, *p*-value = 6e−04; *r* = −0.68, *p*-value = 0.002) (Fig. 6A). Similarly, all three indicators were significantly upregulated in the MTR and FOR groups (Fig. 6B). This revealed that the root and leaf response patterns to *Fusarium oxysporum* infection might differ considerably, with the roots likely more responsive. In contrast, the total SA and SA glucose levels were significantly downregulated in the MTL and FOL groups. GSH-PX, CAT, and free SA synthesis were upregulated, while H_2_O_2_ was significantly downregulated in MTR.

### Identification of TFs involved in FOR and MTR

**Fig. 7:**
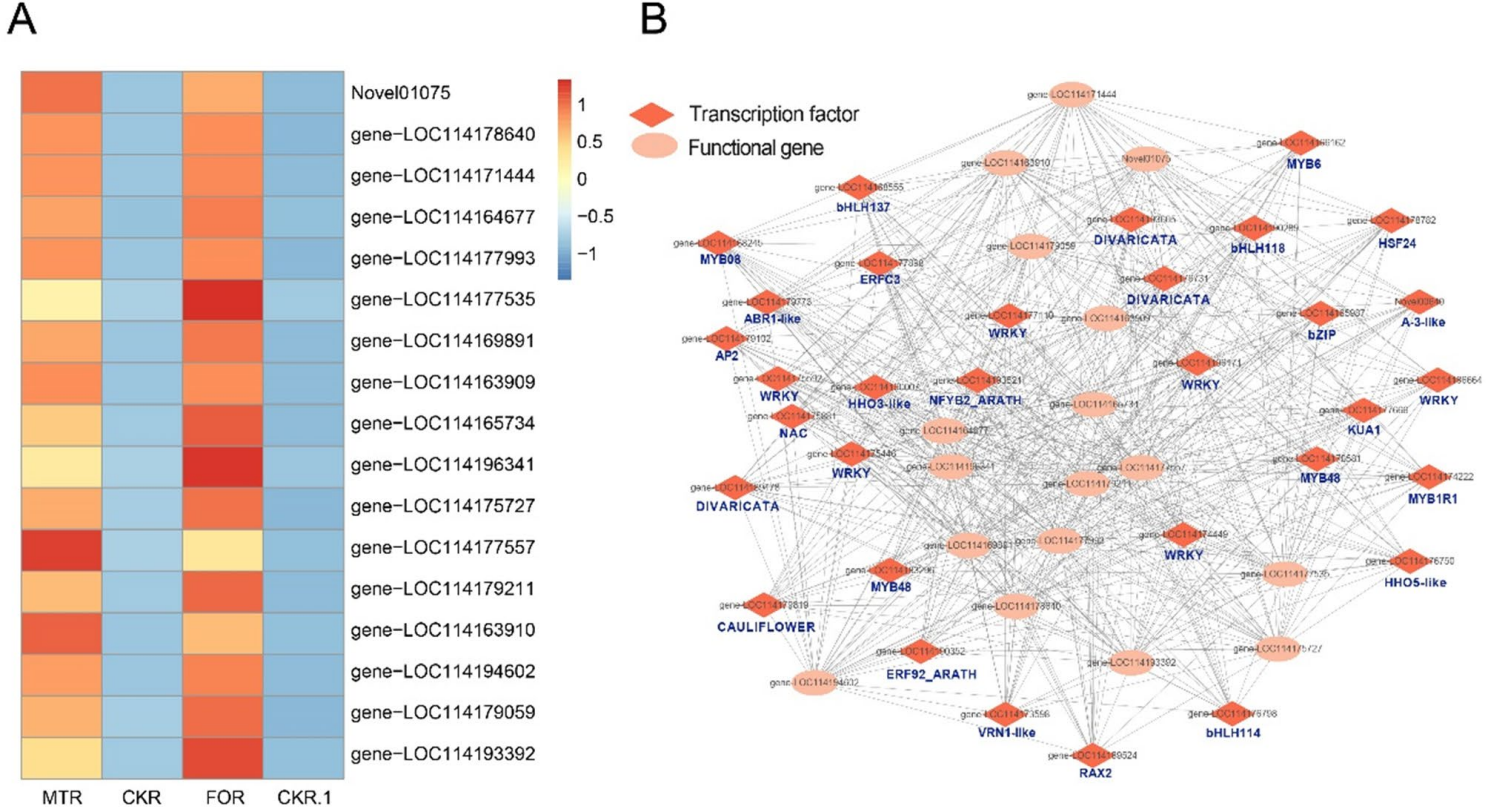
Expression analysis of 51 redox genes in the blue module under FO and MT treatment in roots. (**A**) Heat map of 17 overlap genes under root FO and MT treatment. (**B**) Coexpression network of 17 redox genes and 32 transcription factors

Expression analysis of 51 redox genes from FOR and MTR in the blue module showed that 17 overlapping genes were upregulated (Fig. 7A). Seventeen redox genes and 32 TFs formed a complex interconnected network (Fig. 7B). The TFs families WRKY, MYB, bHLH, ERF, ABR, HSF, NAC, and ZIP were identified in the antioxidant response in the blue module. Interestingly, these 32 TFs were directly related to 17 genes, excluding WRKY46 (gene-LOC114186664), indicating that their expression patterns were highly similar and that they might regulate the expression of these genes during FO infection. Among them were six WRKY- and five MYB-type TFs, indicating that these two types of TFs played highly significant roles in plant stress tolerance. These WRKY-type TFs included WRK17, WRKY23, WRKY29, WRKY46, WRKY65, and WRKY69 (gene-LOC114196171, gene-LOC114175446, gene-LOC114177110, gene-LOC114186664, gene-LOC114175592, gene-LOC114174449); the MYB-type TFs MYB1R1, MYB48-1, MYB6, MYB308, and MYB48-2 (gene-LOC114174222, gene-LOC114183296, gene-LOC114166162, gene-LOC114168245, gene-LOC114170581) also appeared in this module.

### Identification of genes involved in peroxisome metabolism

**Fig. 8:**
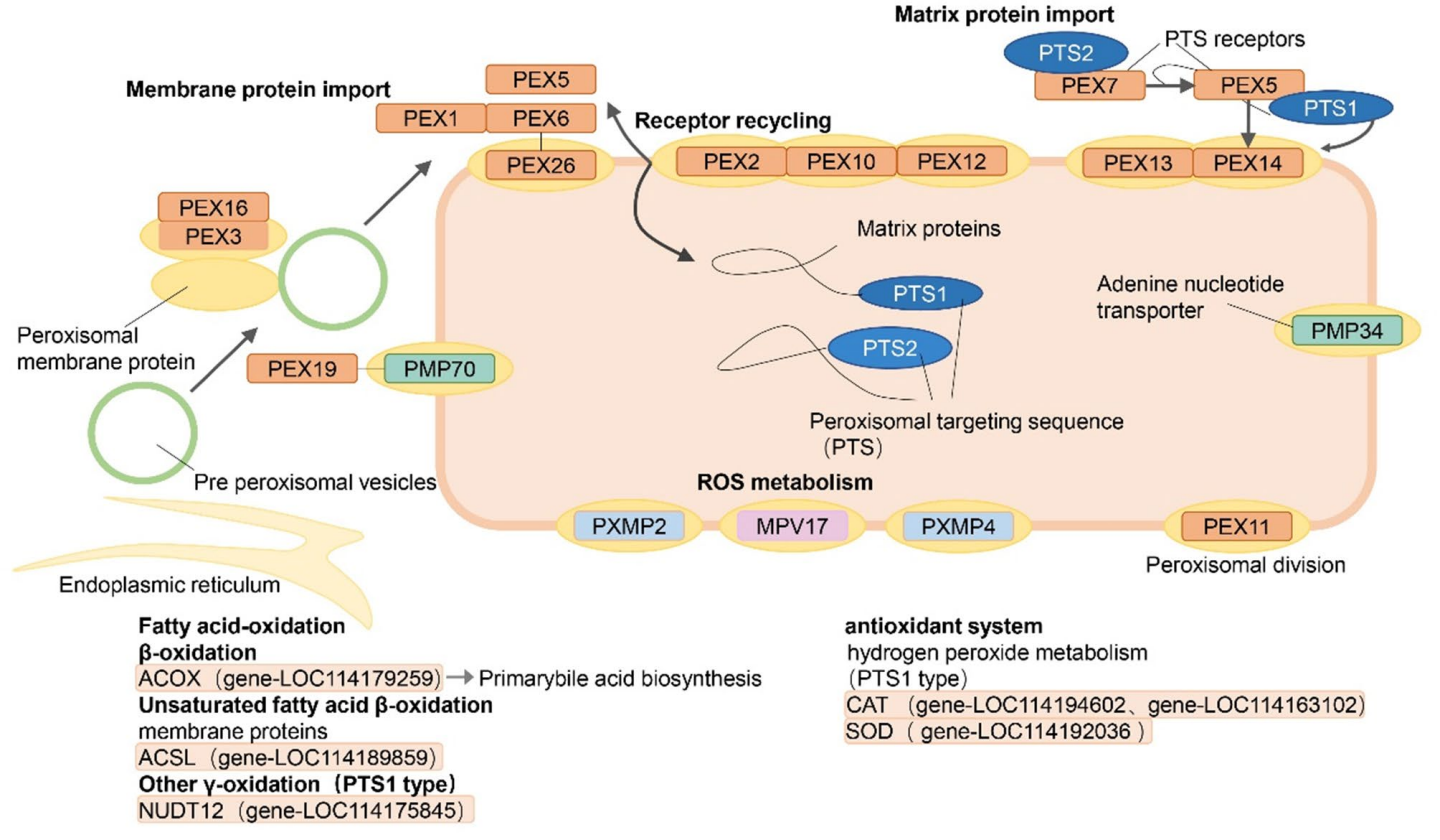
The metabolic pathway of specifically upregulated genes in the peroxisome in MTR. Multiple metabolic pathways occur on the peroxisome, including fatty acid oxidation β-oxidation, unsaturated fatty acid β-oxidation, other γ-oxidation, and hydrogen peroxide metabolism. There are two topological signals in peroxisomal matrix proteins: the C-terminal tripeptide sequence, PTS1-type and the N-terminal PTS2-type, respectively. Hydrogen peroxide metabolism and other γ-oxidation belong to the PTS1-type

The genes involved in peroxisome metabolism were identified using the blue module. SODF and ALDH7 (gene-LOC114192036 and gene-LOC114163305) were significantly upregulated only in the MTR. CAT3, CAT4, NUDT19, SODF, ACOX2, and LACSL1 (gene-LOC114194602, gene-LOC114163102, gene-LOC114175845, gene-LOC114192036, gene-LOC114179259, gene-LOC114189859) were involved in peroxisome metabolism and upregulated in the MTR, which aligned with the KEGG enrichment analysis results. CAT3, CAT4, and SODF were involved in the synthesis of CAT and SOD in the hydrogen peroxide metabolism (PTS1 type) system (Fig. 8). It is the most important free radical-scavenging antioxidant system in plant stress resistance. In addition, NUDT19 was involved in other γ-oxidation pathways (PTS1 type).

### qRT-PCR validation

We randomly selected five genes involved in peroxisome metabolism: PER10, ODBA2, AMO2, NUD19, and SODF (gene-LOC114171444, gene-LOC114164677, gene-LOC114177557, gene-LOC114175845, and gene-LOC114192036, respectively) for validation. The changes in the RNA-seq data were consistent with the relative gene expression trends determined by qRT-PCR, indicating that the RNA-seq data were reliable (Additional file 2: Fig. S3).

## Discussion

*Fusarium oxysporum* is a major causative pathogen of cowpea wilt [31]. When plants are infested with *Fusarium oxysporum*, normal growth and development are affected, and the immune system is activated, causing a series of biochemical reactions [32]. Studies have shown that exogenous inducers can activate plant defence systems. In particular, melatonin, as a natural inducer, is widely used in plants to enhance plant immune [33]. In the present study, we investigated the regulatory mechanisms of melatonin treatment in improving cowpea resistance to *Fusarium oxysporum*.

Melatonin plays autophagic, antioxidant, and immunomodulatory roles in the immune process and is an important regulator of response to pathogenic bacterial infestation [34, 35]. We found that the *Fusarium oxysporum* incidence and disease index of cowpea were significantly reduced at 100 µM MT, with a marked reduction in bacteria-induced plant growth inhibition; however, growth restriction was still observed compared to control plants. Meanwhile, other concentrations of MT exerted insignificant mitigating effects (Fig. 2). The same concentration of melatonin reduces apple damage caused by *Diplocarpon mali* [36]. Indeed, at appropriate concentrations, melation could promote plant root growth and photosynthesis while reducing the osmotic potential to increase plant growth attributes [37]. Moreover, 100 µM melatonin inhibits the growth of pepper anthracnose mycelium, significantly reducing disease symptoms [38]. Therefore, melatonin increased cowpea resistance to *Fusarium oxysporum* in a concentration-dependent manner.

Most secondary metabolites involved in plant defence are produced via the phenylpropanoid pathway [39]. Plants can accumulate secondary metabolites phenolic substances, such as coumaric acid, *p*-coumaric acid, *α*-coumaric acid, ferulic acid, flavonoids, and lignans, at the site of infection, effectively forming a barriers against the pathogen [40]. In the study, the upregulated DEG-enriched pathways in the MTL and MTR included phenylalanine metabolism, tryptophan metabolism, and the biosynthesis of isoquinoline alkaloids, flavonoids, and flavonols, which may produce numerous secondary metabolites involved in plant immunity. Lignin is a secondary metabolite in the phenylpropane metabolic pathway that is directly involved in the plant defence system, acting as a physical barrier to the invasion of pathogenic bacteria into plant cells and their further propagation and spread within the host, spatially confining the pathogen to the site of infection, thus conferring resistance [41–43]. In MTL and MTR, we identified an upregulated POD gene, which encodes a key enzyme involved in lignin synthesis. We also detected upregulated of CYP73A100, 3-O-methyltransferase, and 4-coumarate-CoA ligase in MTL and mannitol dehydrogenase and hydroxycinnamoyl transferase in MTR, which contributes to lignin synthesis and accumulation. Moreover, the expression of BGLU increased in the MTL and MTR. Coumaric acid is a defense metabolite with antifungal activity, and BGLU is an important enzyme in coumarin synthesis [44, 45]. This suggests that exogenous melatonin pretreatment promoted the enrichment of secondary metabolism as well as the expression of defence genes in cowpeas, effectively increasing plant resistance to *Fusarium oxysporum*.

The induction of plant resistance genes occurs primarily at the transcriptional level via TF regulation and is a key aspect of the plant response to biotic stress [46]. Most TFs that responded to *Fusarium oxysporum* under MT conditions were in the WRKY family (Fig. 7). These TFs regulate pathogen-triggered cellular responses in many plants [47] and are induced by phytohormones, such as SA, jasmonic acid, and ethylene. Moreover, WRKY TFs are activated by phosphorylation in response to the MAPK cascade, thus initiating the expression of defence-related genes [48, 49]. For example, WRKY29 and WRKY22 play important roles in *Arabidopsis thaliana* in response to *Botrytis cinerea*, promoting plant resistance to fungal pathogens by regulating downstream signalling pathways in the MAP kinase cascade [50, 51]. Metabolic pathway analysis identified WRKY29 as a defence-related gene that induces the production of phytoalexin accumulation miRNA. Similarly, WRKY29 overexpression increases wheat resistance to Fusarium head blight [52]. Meanwhile, we found that free SA accumulation was significantly higher in cowpea roots. WRKY TFs impact the expression of resistance genes in the SA signalling pathway and positively regulate the ETI response [53]. Therefore, the upregulation of WRKY TFs by MT was likely in response to increased SA accumulation. However, the precise involvement of WRKY TFs in the SA signalling mechanisms in cowpea immunity to *Fusarium oxysporum* requires further investigation.

When plants are exposed to pathogen stress, the most rapid defence response is a burst of ROS, predominantly superoxide and H_2_O_2_ [54]. The accumulation of large amounts of ROS can have toxic effects, causing damage to DNA, RNA, proteins, and membrane oxidation [55]. Melatonin is an effective scavenger that protects plants from ROS and RNS damage and confers environmental stress tolerance [56]. We found increased levels of CAT and GSH-PX and upregulation of CAT3, CAT4, NUDT19, SODF, ACOX2, and LACSL1 gene expression, whereas H_2_O_2_ levels were significantly reduced in the MTR (Fig. 6B). Previous studies have demonstrated that melatonin activates antioxidant redox reactions that trigger the expression of antioxidant enzymes [35]. We found that most of the genes involved in these antioxidant reactions were involved in peroxisomal metabolism (Fig. 8) due to the presence of CAT and GSH-PX in the peroxisome to scavenge H_2_O_2_ [57, 58]. This suggests that MT may increase the antioxidant system and reduce the secondary damage caused by peroxides to plants. In addition, when cowpea plants were infested with *Fusarium oxysporum*, a rapid burst of ROS occurs in the apoplast, chloroplasts, and peroxisomes. The burst of ROS and spraying of exogenous melatonin stimulate the synthesis of endogenous melatonin, triggering a series of MAPK cascades that activate SA synthesis [25, 59]. Hence, MT likely initiated multiple *Fusarium oxysporum* resistance systems in cowpea plants, as evidenced by the numerous types of antioxidant metabolic processes enriched by MT.

## Conclusions

Our study presents evidence supporting the effectiveness of exogenous melatonin application in improving cowpea wilt resistance. Treatment with exogenous melatonin led to the promotion of CAT, GSH-PX, and SA synthesis, as well as a reduction in H_2_O_2_ levels. Exogenous melatonin treatment enriche metabolic pathways related to phenolic synthesis and upregulated the expression of genes involved in lignin synthesis. Furthermore, melatonin treatment activated the expression of TFs, including members of the WRKY and MYB families. These findings can provide important insights into the potential use of exogenous melatonin for enhancing cowpea disease resistance and offer novel strategies for breeding disease-resistant cowpeas.

## Methods

### Plant materials

The susceptible variety ‘Purple Cowpea 6’ was cultivated in the greenhouse at Jiangxi Agricultural University (Nanchang, China). The seeds were grown in plastic pots (90 mm diameter, 120 mm depth) containing peat and vermiculite (2:1 v/v). The plants were cultured under a 14 h light/10 h dark photoperiod, a temperature cycle (25 ℃/20 ℃, day/night), light density 180 ± 10 µmol m^−2^s^−1^, and relative humidity at 70%.

### Isolation and identification of *Fusarium oxysporum*

*Fusarium oxysporum* was isolated from wilted cowpea plants at the planting base of Jiangxi Huanong Seed Industry Co., Ltd. The collected samples were incubated on potato dextrose agar (PDA) medium with 0.3% lactic acid for 5 days at 28 ℃. The colonies were transferred to a new PDA medium using an inoculating needle. After the colonies had grown, 8 mL of sterile water was added to elute the spores, and 30 µL of spore fluid was aspirated for plate incubation. Different colony forms were selected and purified until single spores were obtained. The morphological characteristics of single spores were observed under a microscope and maintained in a PDA medium. DNA was extracted using a Rapid Fungal Genomic DNA Isolation kit (Shenggong, Shanghai, China). PCR was performed to amplify the fragments using universal fungal internal transcribed spacer (*ITS*) primers (Additional file 1: Table S2). Amplified fragments were sent for sequence identification (Tsingke Biotechnology, Beijing, China). The obtained sequences were compared by BLAST on the NCBI online website. The sequences were used to construct a phylogenetic tree using MEGA11.0, with the maximum likelihood algorithm (1000 bootstrap replications).

### Melatonin treatment

Cowpea seedlings at 13 days (second triple-leaf period) were treated with different concentrations (0, 100, 200, and 400 µM) of melatonin once daily for three consecutive days [60]. After the third pre-spraying with melatonin, the roots were irrigated with 30 mL of the spore suspension, and the controls were treated with distilled water. Each experiment contained three replicates.

### Measurement of disease indices

Plants were classified on a 0−5 scale for yellowing and wilting leaves, stunted development, and vascular discolouration symptoms, in combination with symptom incidences of 0%, 10%, 25%, 50%, 75%, and 100%. The incidence rate was calculated as the number of diseased plants/total number of plants × 100. Incidence index = Σ (number of plants at each level × value of level)/(total number of plants × value of the highest level) × 100 [3].

### Determination of biochemical parameters

The cowpea roots and young leaves were subjected to melatonin treatment after 1 dpi with *Fusarium oxysporum* and placed at −80 °C. H_2_O_2_, MDA, CAT, and GSH-PX activities were determined according to the manufacturer’s instructions. All kits were purchased from the Nanjing Jiancheng Bioengineering Institute (Nanjing, China).

### Extraction and determination of SA

Roots and leaves (0.2 g) were ground into a powder in liquid nitrogen at 1 dpi and extracted with 3 mL of 90% methanol. The extract was evaporated by rotation at 45 °C and dissolved with 3 mL of ddH_2_O for 10 min at 80 °C. The obtained solution was used to determine salicylic acid (SA) in the free and bound states. The LC-20AT liquid chromatograph was used under the following analytical conditions: chromatographic column: compass C18 (250 mm × 4.6 mm, 5 µM); temperature: 30 °C; mobile phase: acetonitrile: 0.08% formic acid ultrapure water = 25: 75 (v/v); flow rate: 1.0 mL/min; injection volume: 10 µL [61].

### Transcriptome sequencing

The root and leaf samples of cowpea seedlings pretreated with 0, 100 µM melatonin were collected for transcriptome sequencing after *Fusarium oxysporum* infestation at 1 dpi. Untreated roots and leaves were used as controls. Three biological replicates were used for each treatment group. Total RNA was extracted using TRIzol reagent (Invitrogen, Santa Clara, CA, USA) according to the manufacturer’s instructions. The Agilent 2100 (Agilent Technologies, Santa Clara, CA, USA) was employed to accurately detect RNA integrity and subsequently construct libraries for sequencing on an Illumina sequencing platform. Trimmomatic software was used to reject splice sequences and low-quality reads to obtain clean reads [62]. Clean reads were aligned to the reference cowpea genome (RefSeq: GCF_004118075.1) using Hisat2 [63]. Gene expression levels were assessed using fragments per kilobase per million mapped fragments (FPKM) values; each sample was analysed for gene expression levels using the HTSeq software [64]. Differential gene expression analysis was performed using the R package DESeq. Differential gene thresholds were defined as *p* < 0.05, and fold change > 2.0 [65].

### Gene coexpression network analysis

The relative expression of each gene in the 18 cowpea samples was quantified using the R language WGCNA. The gene set was first filtered for a low coefficient of variation and low gene expression. The samples were then analysed using hierarchical clustering to remove outlier. Soft threshold parameters were selected for neighbourhood matrix weights β-value (β = 1−30) to construct the weighted gene coexpression network. By hierarchical clustering of the topological overlap measure (TOM) matrix, the TOM value served as an indicator for evaluating the level of coexpression of two genes. Core genes were screened based on a Module Membership (MM) > 0.8 and gene significance > 0.3. The top 20 genes were screened as core genes based on the network degree of the key modules. After importing the network into Cytoscape, the plugin cytoHubba was applied to screen data [66].

### qRT-PCR verification

Five randomly selected genes for qRT-PCR to test the reliability of the transcriptome data. Primer 5 software was used to design the primers. The primer sequences are listed in Additional file 1: Table S3. 0.3 g roots and leaves of cowpea were taken from different treatment samples. Total RNA was extracted using a Tiangen RNA Easy Fast Plant Tissue RNA Rapid Extraction Kit. Reverse transcription was performed using a Hifair Yisheng Biology II 1st Strand cDNA Synthesis Kit. Subsequently. qRT-PCR was performed with the Hieff® qPCR SYBR Green Master Mix (Low Rox Plus) on a CFX96 Touch™ real-time fluorescence quantitative RT-PCR system (BioRad, Hercules, CA, USA). The qRT-PCR amplification procedure was as follows: 95 ℃ for 30 s, 95 ℃ for 5 s, 60 ℃ for 30 s, 40 cycles. Each sample was replicated three times and the relative expression was calculated using the 2^-ΔΔCT^ method with *Vu-ubiquitin9* (*VuUBC9*) gene as the internal reference gene [67].

### Statistical analysis

Three replicate trials were conducted for each dataset. Data was assessed using analysis of variance (ANOVA) or *t*-tests with SPSS statistical software.

### Electronic supplementary material

Below is the link to the electronic supplementary material.

**Supplementary Material 1**: Quality statistics of sequencing data and primer information of qRT-PCR

**Supplementary Material 2**: DEG-enriched KEGG pathway scatter plot and validation of DEGs using qRT-PCR

## Data Availability

The transcriptome raw data of the study results were submitted to China National GeneBank DataBase (CNGB, https://db.cngb.org/) under project number: CNP0005303.

## Abbreviations

MT: Melatonin pretreated
FO: Fusarium oxysporum treated
CKL: The control samples in leaves
CKR: The control samples in roots
FOL: Fusarium oxysporum treated samples in leaves
FOR: Fusarium oxysporum treated samples in roots
MTL: Melatonin pretreated samples in leaves
MTR: Melatonin pretreated samples in roots
ROS: Reactive oxygen species
SA: Salicylic acid
DEGs: Differentially expressed genes
WGCNA: Weighted gene coexpression network analysis
