# Supplementary material for: Enhancing cowpea wilt resistance: insights from gene coexpression network analysis with exogenous melatonin treatment
Source: BMC Plant Biol. 2024 Jun 25;24:599. doi: 10.1186/s12870-024-05289-w (PMC11197195; doi:10.1186/s12870-024-05289-w)
Supplement: Supplementary file 1 — Supplementary Material 1: Quality statistics of sequencing data and primer information of qRT-PCR [file 12870_2024_5289_MOESM1_ESM.docx]

Additional file 1: Quality statistics of the sequencing data and primer information of Fungal internal

| transcribed spacer and qRT-PCR  **Table S1** Quality statistics of sequencing data | | | | | | | |
| --- | --- | --- | --- | --- | --- | --- | --- |
| **Sample name** | **Raw reads** | **Clean reads** | **Clean bases** | **Error rate (%)** | **Q20 (%)** | **Q30 (%)** | **GC content (%)** |
| CK1L | 43530392 | 43264998 | 6.49G | 0.03 | 97.70 | 93.17 | 45.08 |
| CK2L | 46674094 | 46358328 | 6.95G | 0.03 | 98.03 | 94.05 | 45.01 |
| CK3L | 38525198 | 38279224 | 5.74G | 0.03 | 98.03 | 94.02 | 45.06 |
| FO1L | 53470224 | 53149614 | 7.97G | 0.02 | 98.16 | 94.38 | 45.5 |
| FO2L | 46884038 | 46586548 | 6.99G | 0.03 | 97.75 | 93.27 | 45.29 |
| FO3L | 42007370 | 41608702 | 6.24G | 0.03 | 97.76 | 93.53 | 45.67 |
| MT1L | 45698034 | 45320994 | 6.8G | 0.03 | 97.79 | 93.51 | 45.89 |
| MT2L | 53701804 | 53281966 | 7.99G | 0.02 | 98.19 | 94.55 | 45.66 |
| MT3L | 44479824 | 44207582 | 6.63G | 0.03 | 97.70 | 93.21 | 45.66 |
| CK1R | 48619738 | 48363984 | 7.25G | 0.03 | 98.03 | 94.11 | 44.89 |
| CK2R | 40323546 | 40067626 | 6.01G | 0.03 | 97.72 | 93.34 | 44.82 |
| CK3R | 62028892 | 61671830 | 9.25G | 0.03 | 97.81 | 93.51 | 44.86 |
| FO1R | 48836018 | 48491894 | 7.27G | 0.03 | 97.79 | 93.56 | 44.78 |
| FO2R | 47594116 | 47237282 | 7.09G | 0.03 | 98.06 | 94.21 | 44.6 |
| FO3R | 41833432 | 41641248 | 6.25G | 0.03 | 97.87 | 93.66 | 45.0 |
| MT1R | 54385512 | 53931648 | 8.09G | 0.03 | 97.87 | 93.78 | 44.76 |
| MT2R | 53659810 | 53325254 | 8.00G | 0.03 | 97.74 | 93.38 | 45.01 |
| MT3R | 48038294 | 47756148 | 7.16G | 0.03 | 97.46 | 92.69 | 44.84 |

**Table S2** Fungal internal transcribed spacer primer

| **Gene** | **Forward primer sequences (5′–3′)** | **Reverse primer sequences (3′–5′)** |
| --- | --- | --- |
| ITS 1 | TCCGTAGGTGAACCTGCGG | \ |
| ITS 4 | \ | TCCTCCGCTTATTGATATGC |

**Table S3** Primer information of qRT-PCR

| **Gene** | **Forward primer sequences (5′–3′)** | **Reverse primer sequences (3′–5′)** |
| --- | --- | --- |
| PER10 | TGGATGCCGTGACTACAAACA | GCAGTGTCACCCAAAAGAGC |
| ODBA2 | TGAAGTGCAGAGGCAAGGTAG | TGTTGCAGTGTAAAACCACGC |
| AMO2 | GCTGTGGAGTATGCTGAGTGT | GGCTAGGAGCATCAGCTTCG |
| NUD19 | CCCAGCAAACTCCGGTATCT | GTAGCCCAATTCTCGCGTTG |
| SODF | GATGAGCCAGCAGACACTCG | AGCTGGAAGAATGTCACCCT |
| Vu-ubiquitin 9 | ACTCACTATCTCCAAGGTACT | GCCAGCCATTCTTCAATATAC |
