# Supplementary material for: Enhancing cowpea wilt resistance: insights from gene coexpression network analysis with exogenous melatonin treatment
Source: BMC Plant Biol. 2024 Jun 25;24:599. doi: 10.1186/s12870-024-05289-w (PMC11197195; doi:10.1186/s12870-024-05289-w)

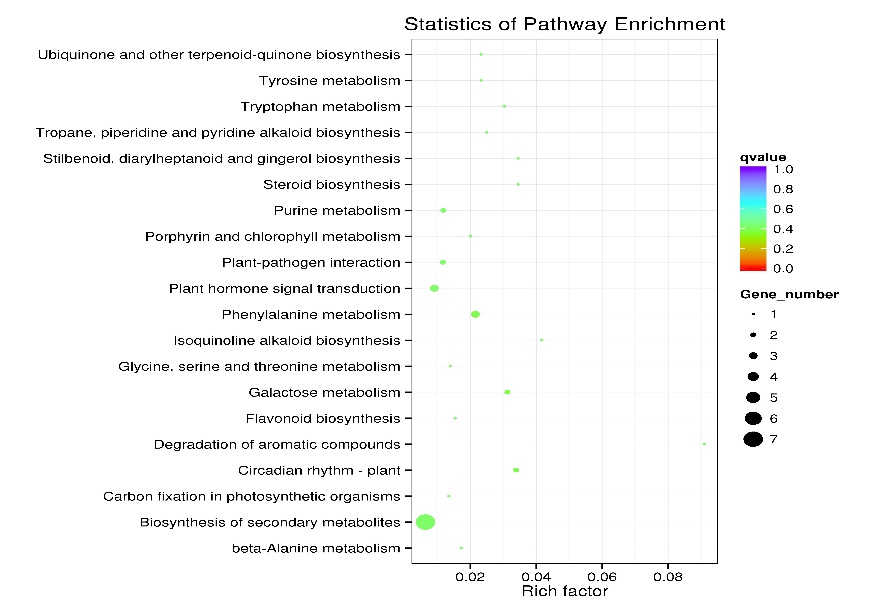
Additional file 2: DEG-enriched KEGG pathway scatter plot and validation of DEGs using qRT-PCR

Fig. S1 MTL vs FOL DEG enriched KEGG pathway scatter plot


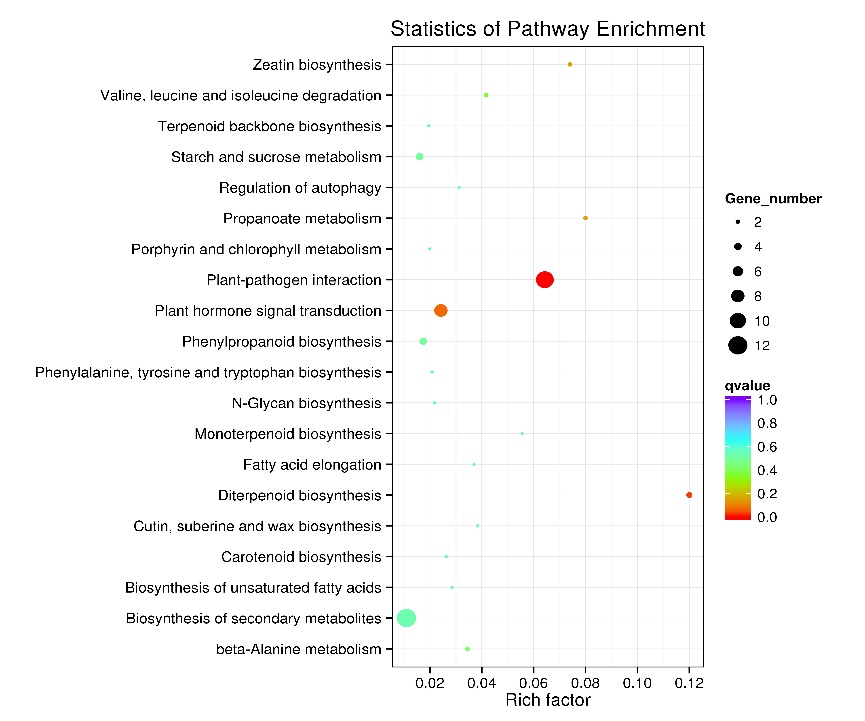


Fig. S2 MTR vs FOR DEG enriched KEGG pathway scatter plot

Fig. S3 Validation of DEGs by qRT-PCR. The bar chart shows the FPKM values of genes in RNA-seq, and the line chart shows the relative expression levels measured by qRT-PCR.


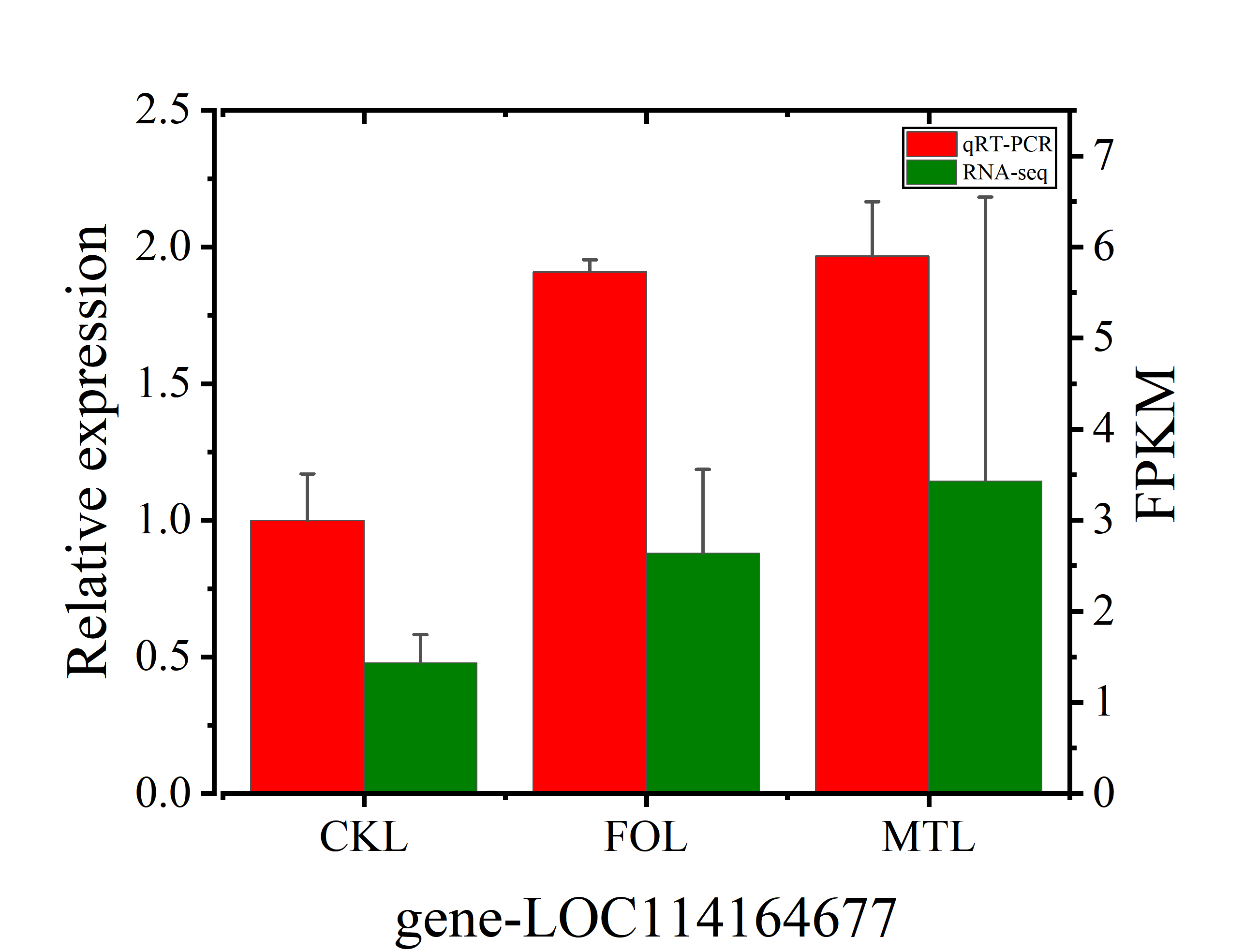

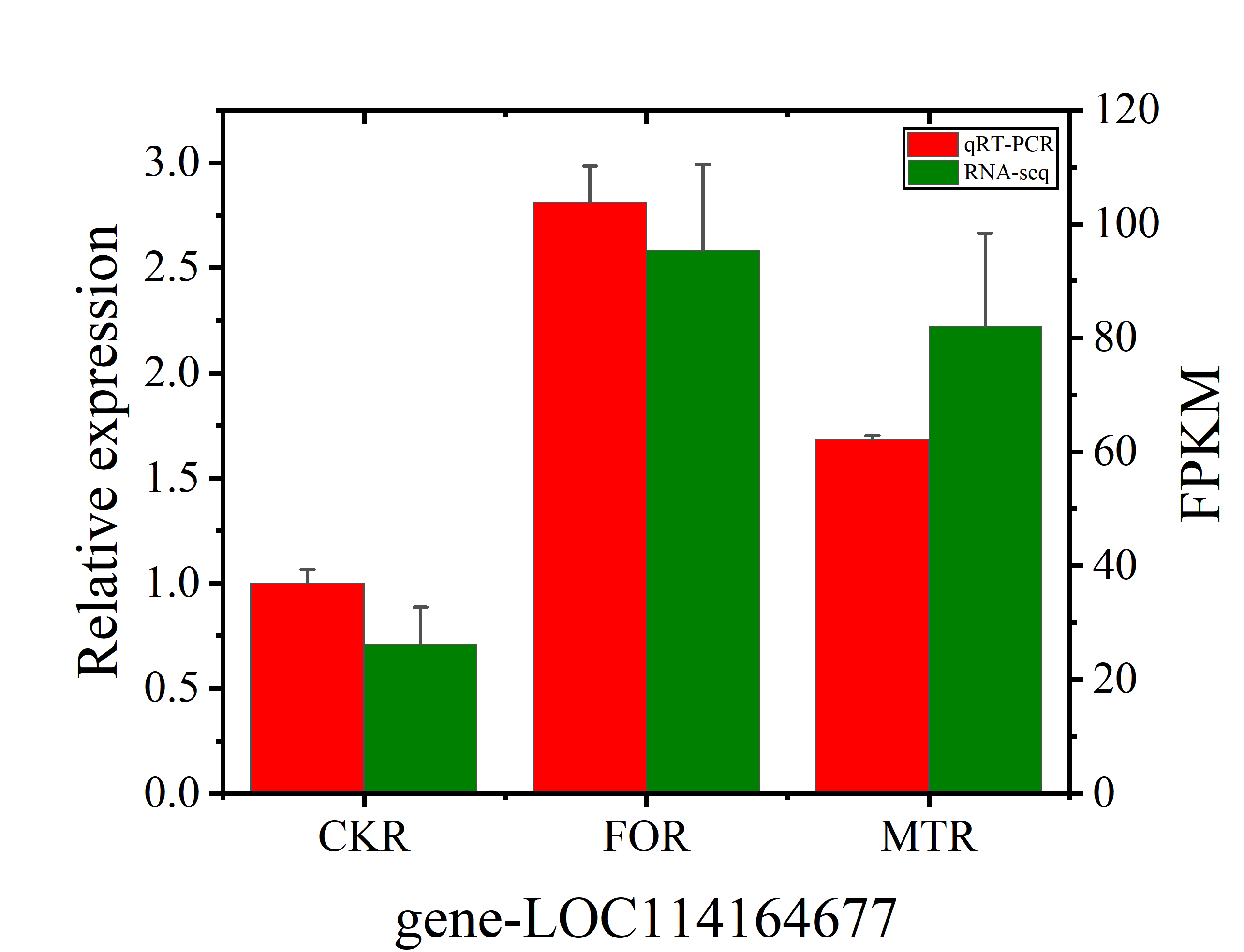

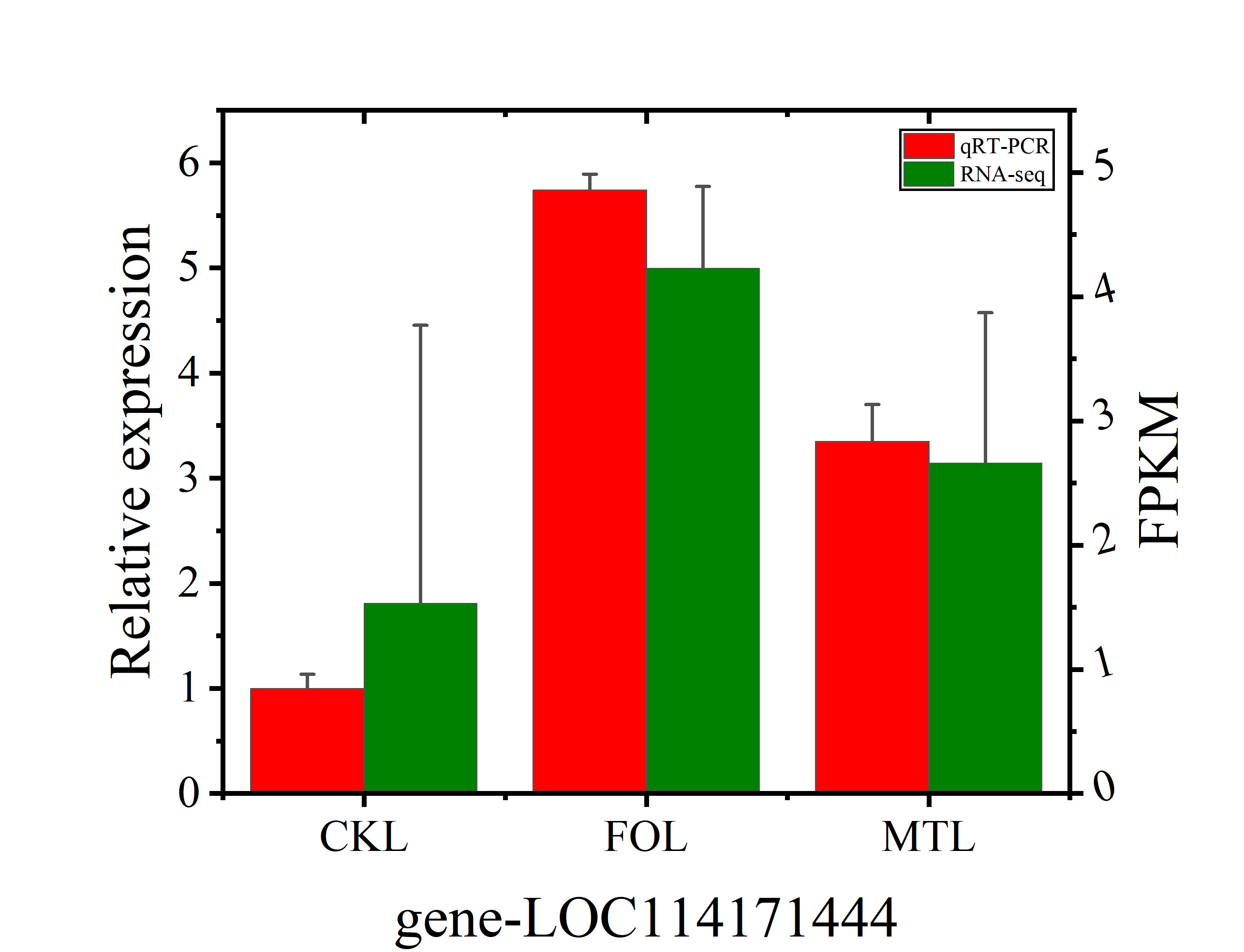

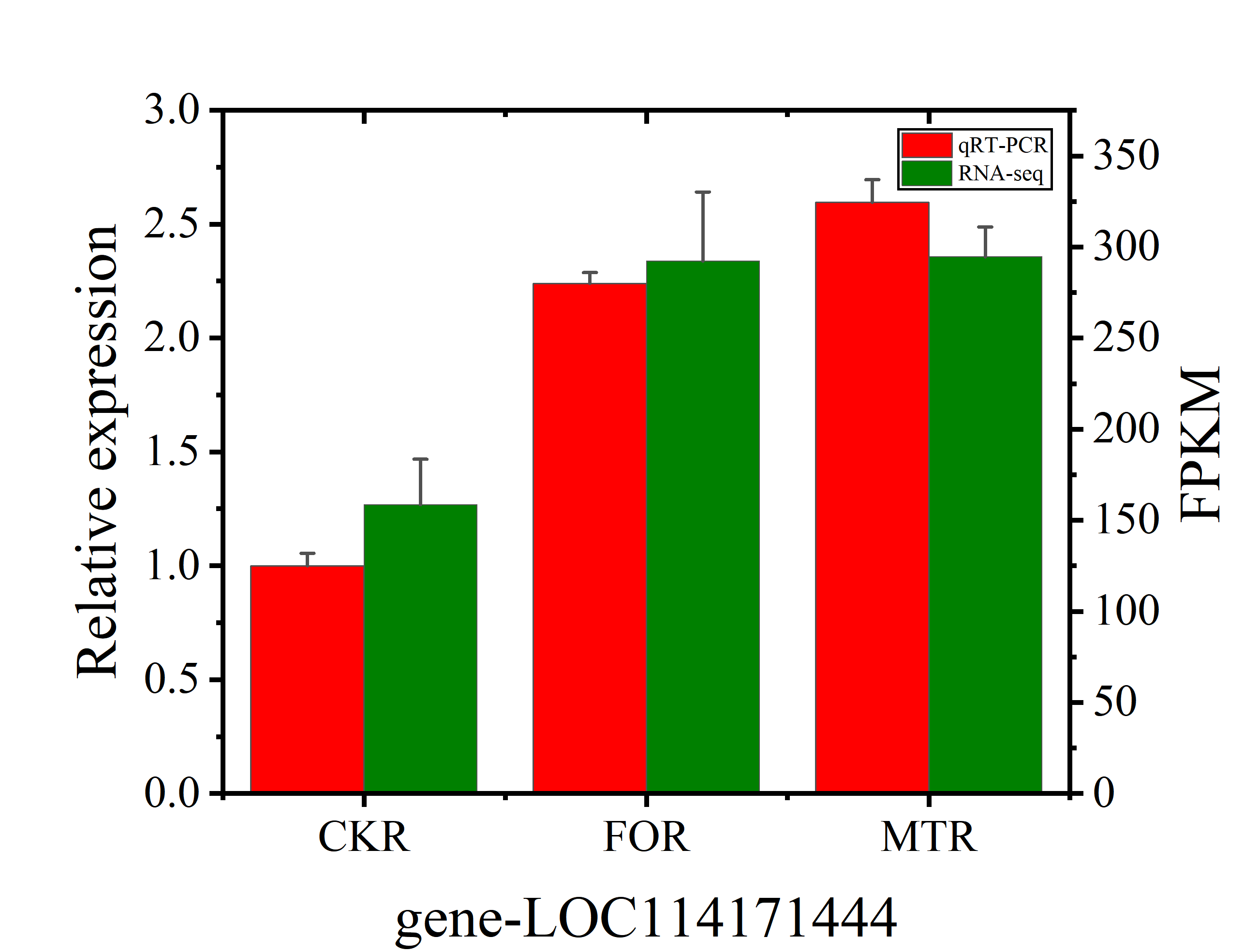

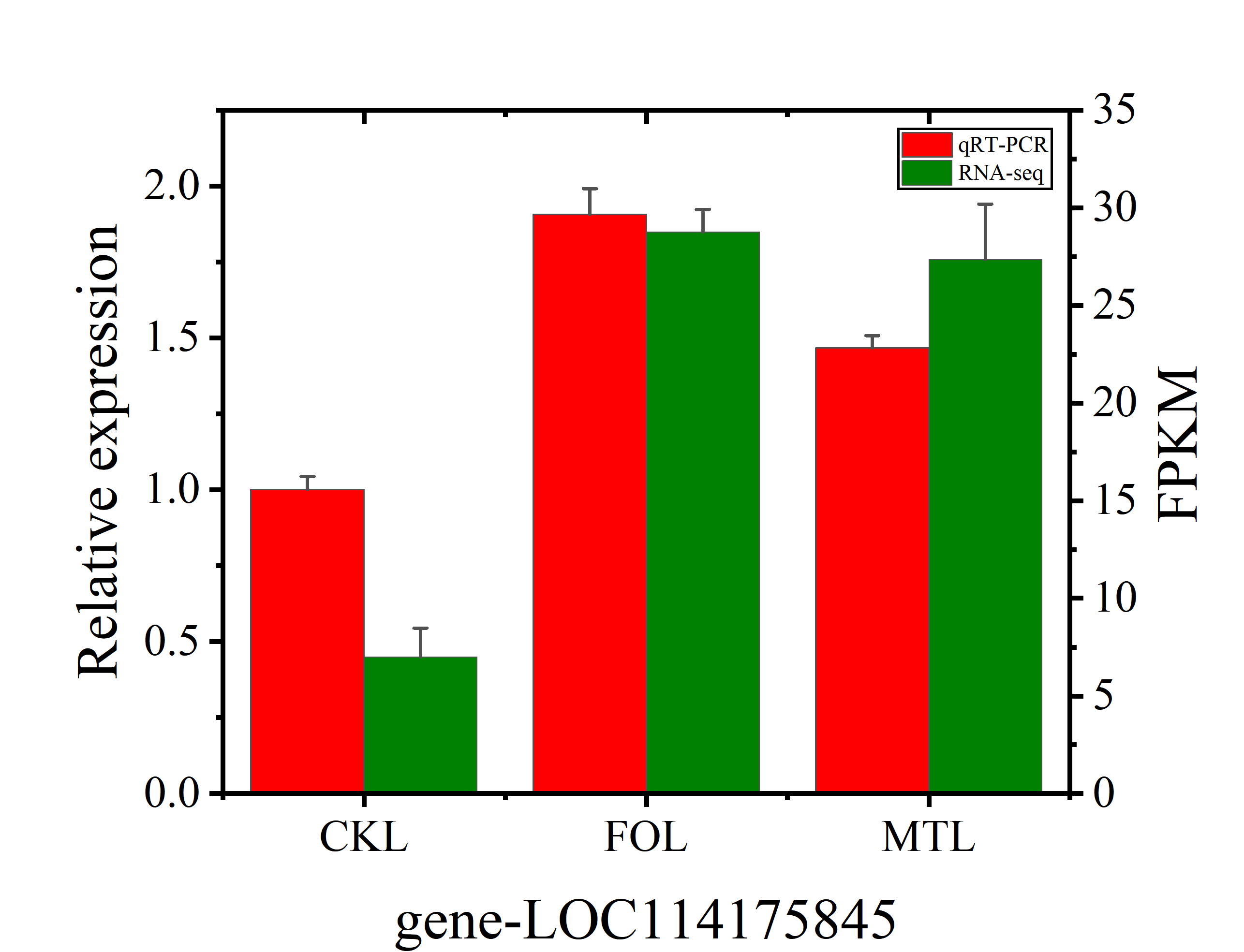

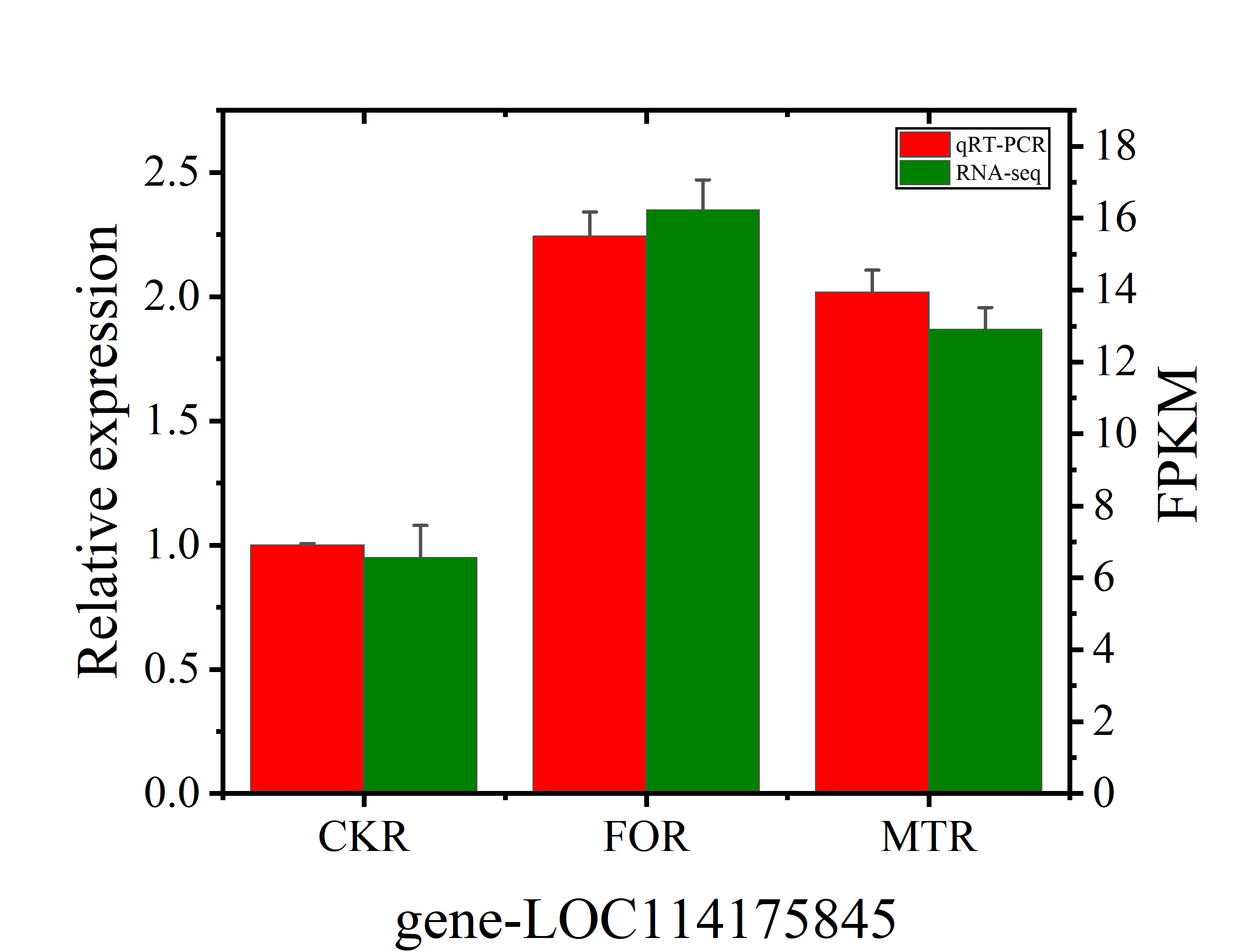

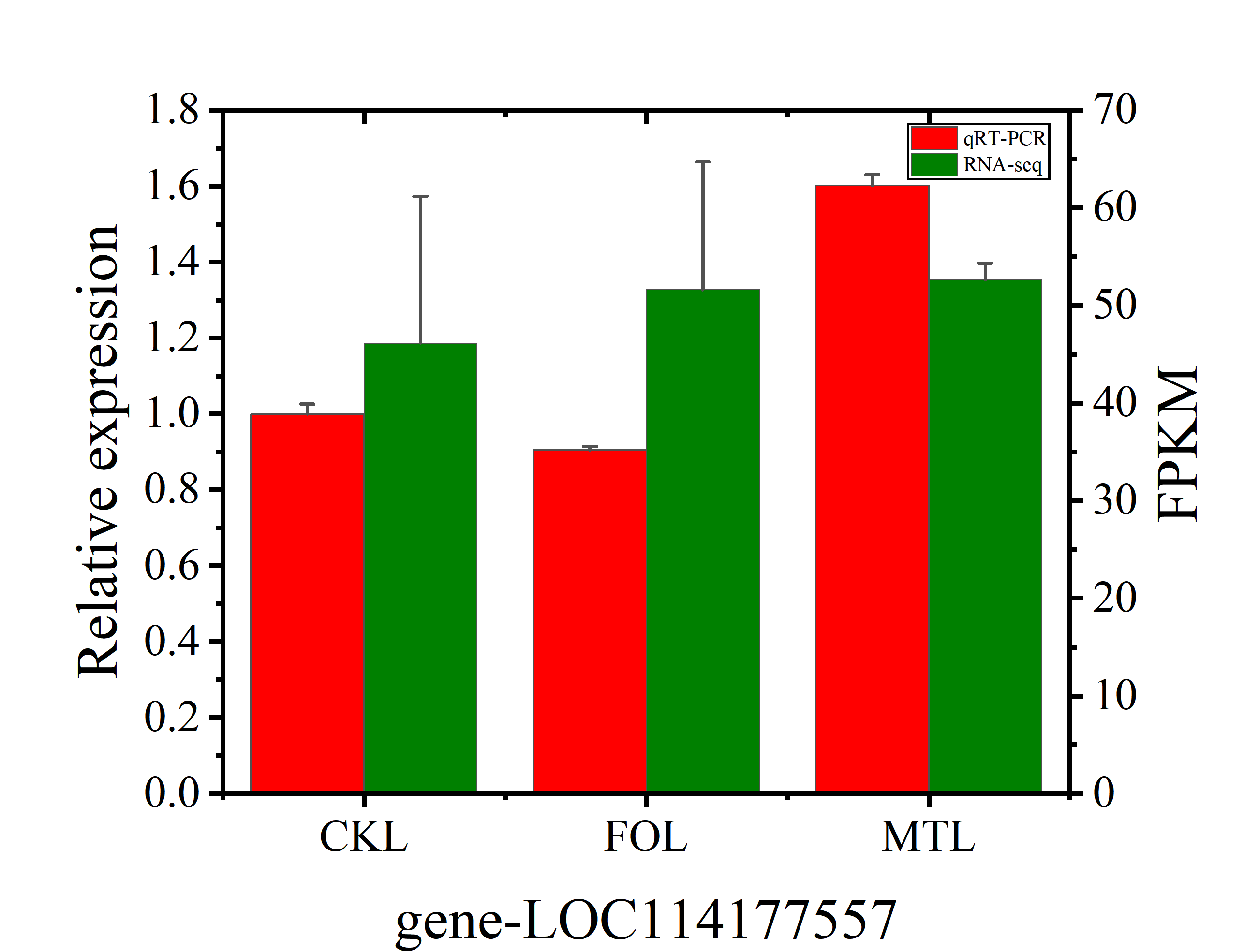

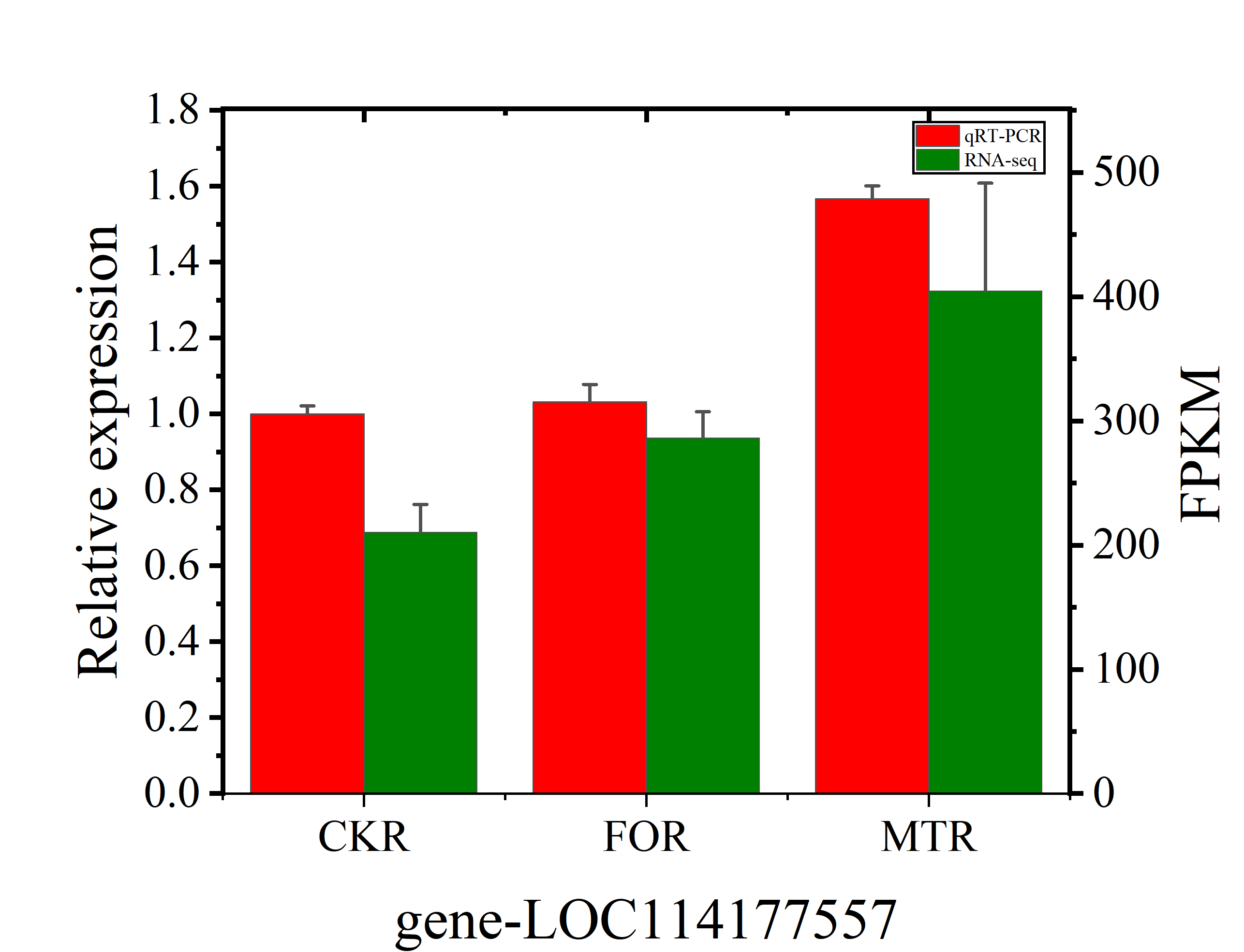

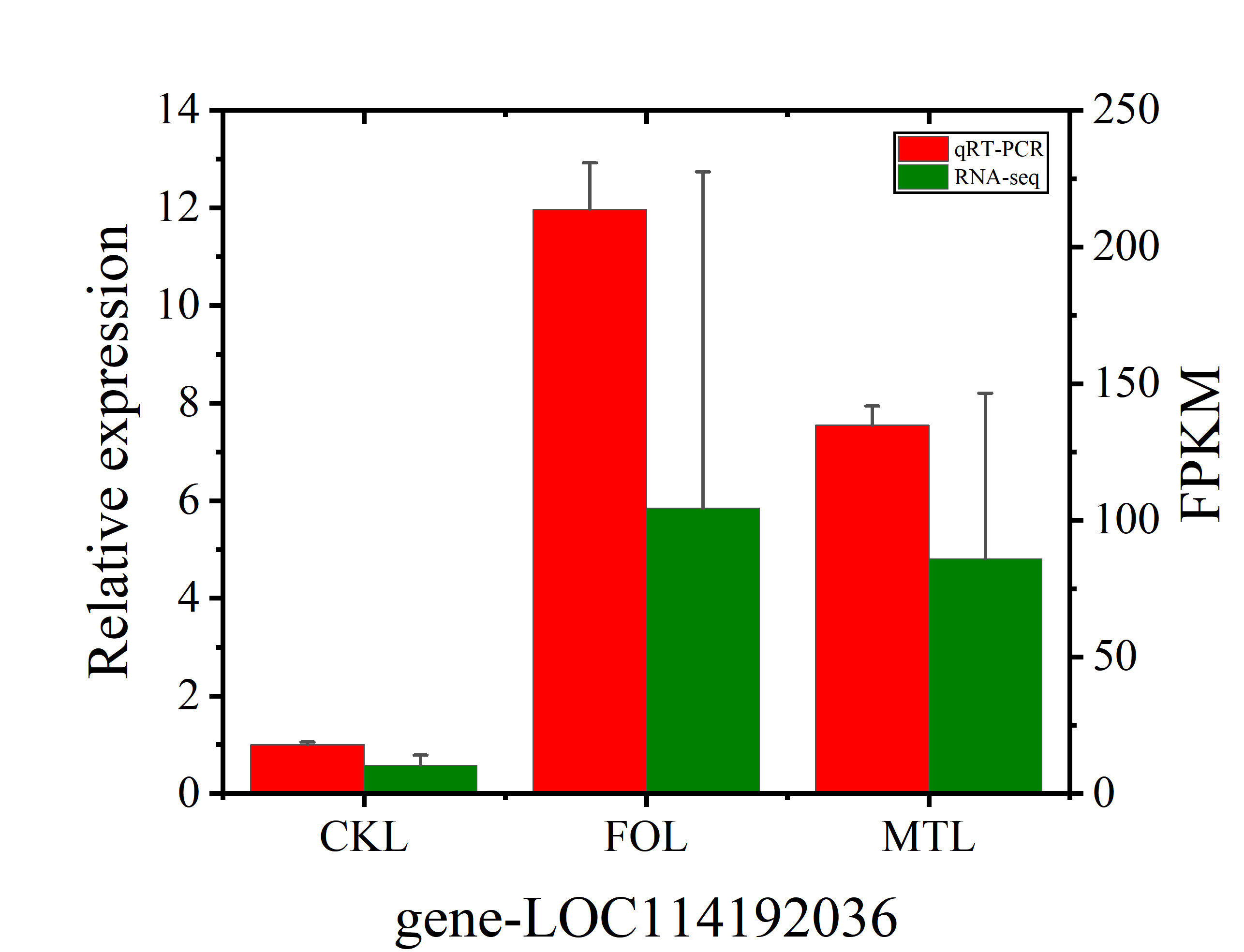

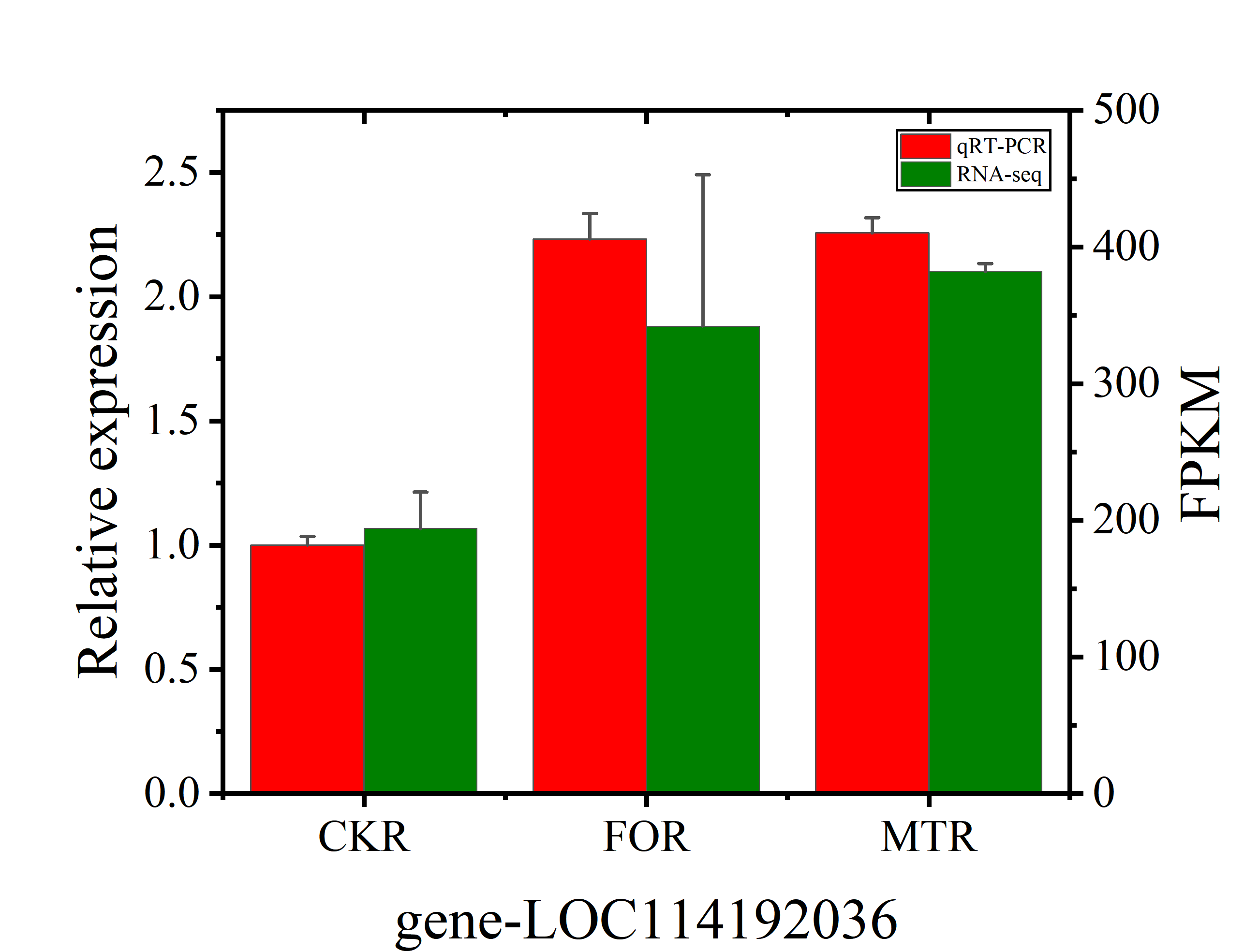

Supplement: Supplementary file 2 — Supplementary Material 2: DEG-enriched KEGG pathway scatter plot and validation of DEGs using qRT-PCR [file 12870_2024_5289_MOESM2_ESM.docx]
